# Mesenchymal Stem Cell Fate: Applying Biomaterials for Control of Stem Cell Behavior

**DOI:** 10.3389/fbioe.2016.00038

**Published:** 2016-05-13

**Authors:** Hilary J. Anderson, Jugal Kishore Sahoo, Rein V. Ulijn, Matthew J. Dalby

**Affiliations:** ^1^Centre for Cell Engineering, University of Glasgow, Glasgow, UK; ^2^Department of Pure and Applied Chemistry, Technology and Innovation Centre, University of Strathclyde, Glasgow, UK; ^3^Advanced Science Research Centre (ASRC), Hunter College, City University of New York, New York, NY, USA

**Keywords:** mesenchymal stem cells, bioengineering, materials synthesis, nanotopography, stimuli-responsive material

## Abstract

The materials pipeline for biomaterials and tissue engineering applications is under continuous development. Specifically, there is great interest in the use of designed materials in the stem cell arena as materials can be used to manipulate the cells providing control of behavior. This is important as the ability to “engineer” complexity and subsequent *in vitro* growth of tissues and organs is a key objective for tissue engineers. This review will describe the nature of the materials strategies, both static and dynamic, and their influence specifically on mesenchymal stem cell fate.

## Introduction

The materials engineering field encompasses various techniques allowing the application of smart materials to tissue engineering (TE). TE can utilize these materials as either a scaffold to support cells *in vivo* or as an enabling technology to improve cell growth and differentiation *in vitro* (Murphy and Atala, 2013). TE has been applied to a range of organs, including the bladder (Atala et al., 2006) and trachea (Macchiarini et al., 2004), which have been used clinically. The knowledge and experience gained from these studies will enable the construction of organs of greater complexity and higher order architecture, e.g., the heart (Hoerstrup et al., 2000; Ott et al., 2008). In the future, the synthesis of organs in the lab potentially allows for the creation of “off the shelf” constructs that may alleviate the need for donors and complex surgeries (Kode et al., 2009). However, there are some limitations to the progress of this field, including the ability to precisely control growth and differentiation of stem cells. Stem cells are well placed to underpin TE due to their unique characteristics of self-renewal and differentiation. This feature of stem cells can address the requirement of complexity in TE, i.e., multiple tissue organs from a single cell source. It would, however, require precise organization of directive cues throughout a scaffold and ideally these cues should be presented only when required (i.e., introducing space-time control). In other words, producing man-made mimics that copy key features of extracellular matrix (ECM) and more specifically the stem cell niche is a worthwhile, albeit challenging endeavor with potential clinical and socioeconomic benefits (Oreffo et al., 2005).

Stem cells are non-specialized cells with the ability to differentiate (become other cell types) or self-renew (replicate without differentiating). To exploit the cells *in vivo*, scaffolds must be made from materials that are ideally bioactive, biodegradable, and biocompatible in order to replicate key features of the ECM. Polymers, such as polyglycolic acid (PGA), polylactic acid (PLA), and polyethylene glycol (PEG) make ideal scaffolds as they are biocompatible and FDA (Food and Drug Administration) approved (Koh and Atala, 2004). The first event of key importance upon stem cell interaction with materials is adhesion. Adhesion to the substrate is imperative as stem cells are anchorage-dependent meaning that those unable to adhere will apoptose via anoikis (“homelessness”) (Dalby et al., 2014). Initial control of stem cell adhesion to biocompatible scaffolds ensures cell survival, then a differentiation cue can be provided to generate a desired cell population.

**Figure 1:**
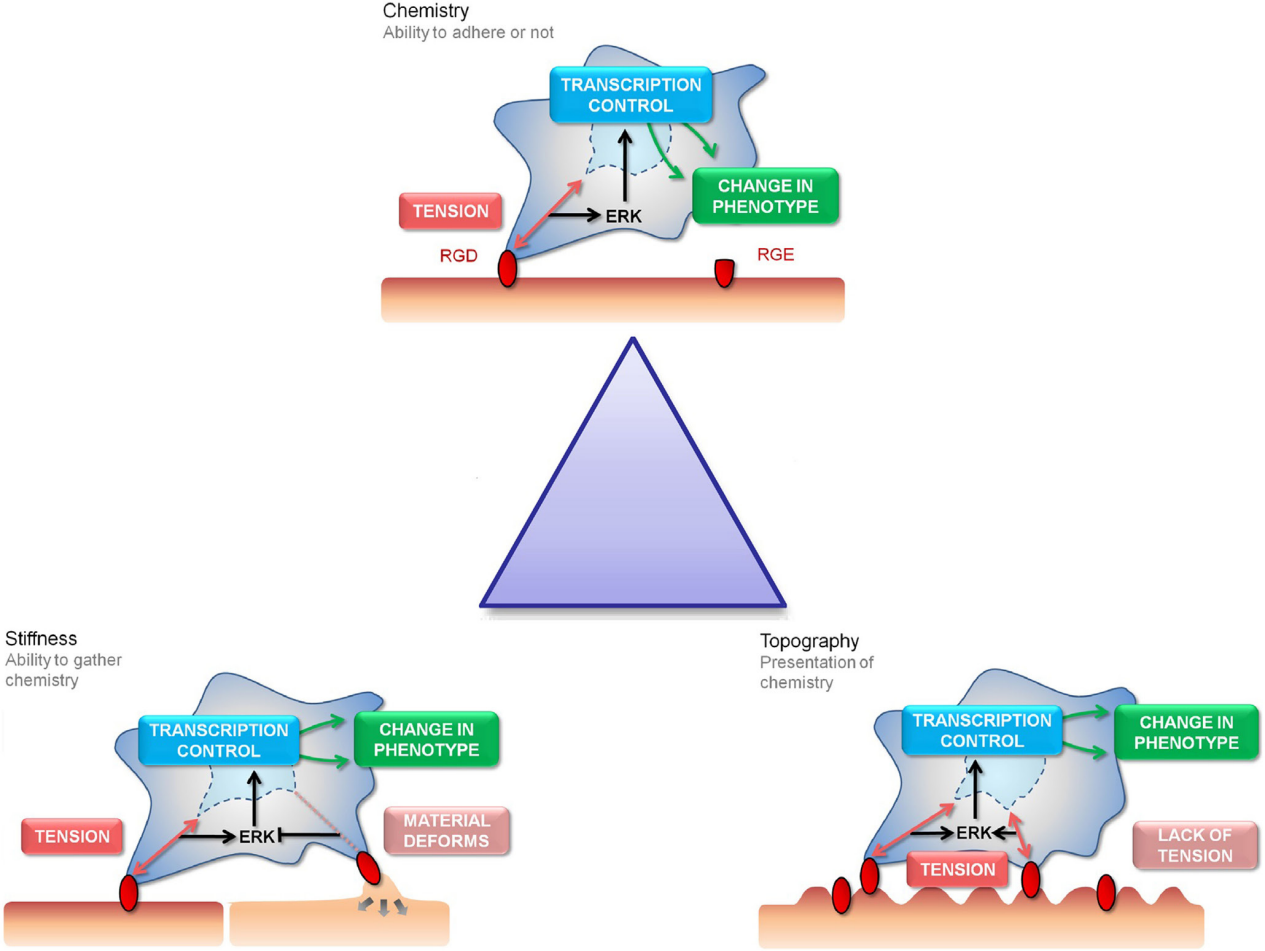
**The “Triangle” of material/surface interface**. The “triangle” of materials refers to variations in chemistry, stiffness, and nanotopography to control the interactions with MSC focal adhesions. The three cartoon panels show MSC adhesion to chemical, stiffness, and topographically modified surfaces and how this influences stem cell tension and signaling and, hence, subsequent differentiation and phenotype (as will be discussed). The cells are shown to extend filopodia to “find” adhesion ligands (shown in red). Binding of the cell through focal adhesions to these ligands creates tension and activates signaling. Chemical functionality can be used to fabricate areas of high adhesion (red) or low adhesion (capped in green) for the cells to respond to (the cell is shown in a 3D scaffold). Stiffness will affect the cells ability to produce tension through focal adhesions formation (the cell is shown on a planar surface). Topography will present the adhesion ligands to the cells in either a favorable or unfavorable manner, again affecting adhesion and subsequent tension and signaling (the cell is shown on a 2D surface).

Biomaterials have evolved rapidly over the last 30 years. Originally, first-generation materials purposed for biocompatibility and mechanical integrity gained popularity. This progressed to understanding that materials could be bioactive, eliciting desired cell response and could also be biodegradable with the aim of being replaced with native tissue after the support and templating role was complete; second-generation biomaterials included hydroxyapatite and bioglasses. There is a drive toward third-generation materials where reproducible molecular control of cells is targeted, activating the genome to regenerate the tissue (Hench and Polak, 2002). Such materials could be powerful tools for stem cell bioengineering as we start to manipulate biochemical control of stem cell fate and function (Oreffo et al., 2005). It is necessary to create and enhance existing technologies due to the limitations of existing culture methods. For example, tissue culture plastic, which has served well for somatic cells, is far from ideal to expand the stem cell population as niche cues that regulate self-renewal are missing. Therefore, there is a need to introduce new technologies that provide a stimulus to direct stem cell behavior in a user-defined manner (Lutolf and Blau, 2009; Lutolf et al., 2009). Attempts to improve cell culture methods have centered on the manipulation of three key materials features: topography, stiffness, and surface chemistry (Figure 1). Each example has provided more information on the nature of mesenchymal stem cell (MSC) adherence, growth, and differentiation.

## Stem Cell Characteristics

Stem cell self-renewal can be symmetrical where two stem cells are produced to enrich the stem cell population or asymmetrical where a stem cell and a progenitor cell is produced, hence responding to regenerative demand and maintaining stem cell number. Progenitor cells migrate from the niche expanding in number (transit amplification) and become more specialized as they progress from stem cell to progenitor cell to differentiated cell (Watt and Hogan, 2000). Stem cells have an extended capacity for self-renewal due to constitutive telomerase activity whereas terminally differentiated cells are subject to senescence. Furthermore, adult stem cells often use quiescence as a tactic to avoid DNA damage when they are not active (Watt and Hogan, 2000).

Adult stem cells, while able to self-renew, have a defined differentiation potential and only form cell types within a lineage range – usually to replenish cells in the area local to the niche that controls the stem cells (Heissig et al., 2002). For example, MSCs are derived from the mesenchyme layer in the developing embryo and form tissues derived from that layer, including bone and fat (Pittenger, 2008). MSCs are attractive as an autologous therapy source as they are ethically sourced and it is simple to obtain the cells from bone marrow, i.e., removal of bone marrow from hip replacement surgery for lab use or use of iliac crest aspiration or lipoaspiration. There is also evidence that MSCs are immune-modulatory as they lack the major histocompatibility complex (MHC) Class II, indicative of evasion of the immune system. Furthermore, they can reduce expression of inflammatory dendritic cells and suppress effector T cells making MSCs a candidate for allogeneic as well as autologous treatments (Kode et al., 2009).

Currently, the exact factors that stem cells require to differentiate *in vivo* are unknown. What is recognized is that the stem cell environment, the niche, is an important factor for the regulation of behavior. The niche is a 3D microarchitecture that incorporates many cell types supported by an ECM made of proteins, including collagen and fibronectin (Ehninger and Trumpp, 2011). It is not only the niche microenvironment that influences the cells but secreted factors of other cell types also have regulatory effects (Hartmann, 2006).

The ECM is required not only for structural support but also provides substrate-specific ligands for migration, adhesion, proliferation, and function in addition to chemical and physical signals to regulate many aspects of the body’s physiology (Visse and Nagase, 2003). The niche is dynamic and complex and it is, thus, unsurprising that the cells lose control of self-renewal and spontaneously differentiate when plated on tissue culture plastic (Lutolf and Blau, 2009). It is possible that learning from nature, replicating an aspect of the native system that is robust enough to be engineered and synthesized, could help us not only to develop scaffolds that direct differentiation as desired but also surfaces that could control growth of quality stem cells.

## Cell–Surface Interaction

To interact with the ECM, cells use receptors such as integrins that ligate to specific peptide motifs within the ECM (Geiger et al., 2001). Each ECM protein has characteristic motifs within its sequence, for fibronectin; RGD and LDV (Yamada, 1991), for laminin; IKLLI, IKVAV, PDSGR, and YIGSR (Weber et al., 2007), for collagen; DGEA (Weber et al., 2007). Each sequence is recognizable by different cell receptors, namely integrins. Integrins are the principal family of receptors that mediate cell adhesion. Consisting of α and β subunits, forming a dimer to interact with the dynamic presentation of ECM proteins. The differing combination of α and β subunits allows ligand specificity for a particular motif (Hersel et al., 2003), for example, α_5_β_1_ integrin binds to an RGD ligand, in addition, other integrin motifs that bind (although not limited to) RGD include most α_v_ combinations, α_8_β_1_ and α_IIb_β_3_ (Humphries et al., 2006). With the diversity of ECM motifs and the possibility of a number of integrin conformations to interact with, this has a direct impact on the type of cell–ECM interaction and subsequent cell behavior. Undoubtedly, the most characterized feature of the ECM is the sequence arginine, glycine, and aspartic acid (RGD) (sometimes lengthened with a serine residue to RGDS) often described as the cell adhesive peptide (Ruoslahti and Pierschbacher, 1987). This sequence is not limited to fibronectin and is incorporated into various ECM proteins, such as collagen, vitronectin, and osteopontin. We have chosen RGD as the focus of this review due to the preferential use in the biomaterials engineering. The aforementioned ECM motifs, while an intrinsic part of ECM interactions, are beyond the scope of this article.

Ligand-occupied integrins stimulate formation of focal adhesions (FAs) whereby integrin receptors cluster and recruit other proteins, including cytoskeletal elements to establish a connection between the cell and the ECM. It has been shown that FA formation is determined and limited by spacing between integrins driven by ECM ligand availability. Cavalcanti-Adam demonstrated this by tethering RGD to gold nanoparticles at pre-determined distances of either 58 or 108 nm, at 58 nm the cells spread and adhere to the particles after 3 h. In comparison, at 108 nm cell morphology remains rounded after 24 h. The spacing of 108 nm was beyond the optimal spacing for integrin gathering; therefore, adhesion, FA formation, and cell spreading were prevented (Cavalcanti-Adam et al., 2007). Structurally, the FAs act as an internal scaffold, their size is dependent on the number of actin fibers available to gather together (a direct result of binding ECM).

**Figure 2:**
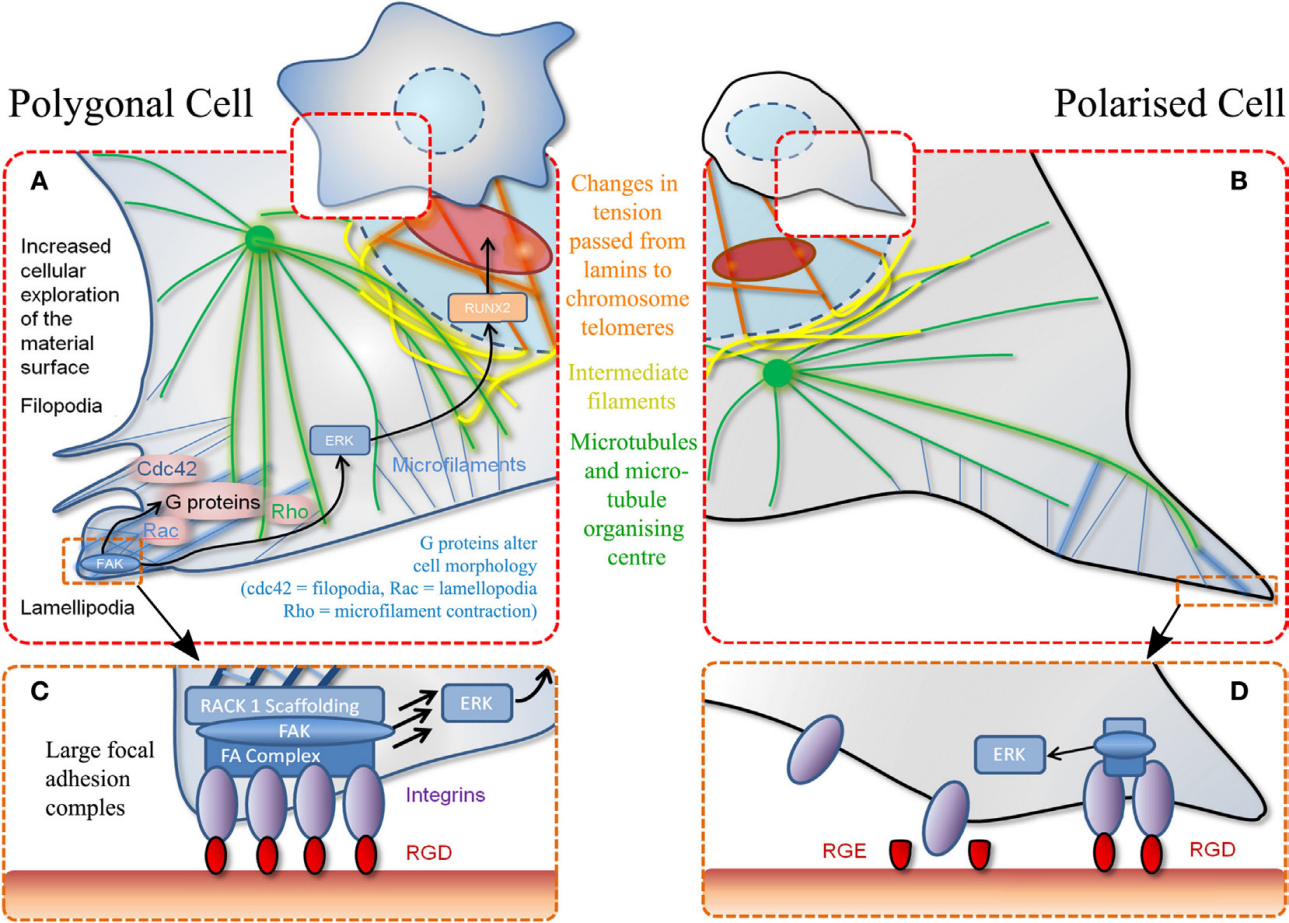
**Schematic of MSC adhesion**. **(A)** Binding to material surface by lamellipodia stimulates a signaling cascade. This results in transcription factor expression (RUNX2) that stimulates differentiation via other G-proteins and effectors. Polygonal cells adhere to a material at various positions encouraging cell spreading and decreasing motility. **(B)** Polarized cells refer to adhesion at a single point through the same mechanisms described in **(A)**. **(C)** Increased magnification of adhesion event described in **(A)** whereby a favorable adhesion motif (RGD) is found in high density. **(D)** Magnification of adhesion event described in **(B)** whereby adhesion motif (RGD) is found in isolation. Also demonstrates non-adhesiveness of a closely related RGE (aspartic acid replaced with glutamic acid) peptide.

Direct mechanotransduction is the process by which cells turn adherent stimuli into a cellular response (creation of FA and maintenance of stress fibers) capable of directly manipulating chromatin, altering gene expression and, therefore, cell behavior (Tsimbouri et al., 2013). Indirect mechanotransduction describes biochemical cascades that are the result of cellular adhesion *via* activation of focal adhesion kinase (FAK) and mitogen-activated protein kinase (MAPK) mediated by G-proteins, such as Rac, Cdc42, and Rho (Figure 2). Rho belongs to the Ras superfamily and is responsible for the regulation of FA and stress fibers. Other G-proteins involved in cytoskeleton arrangement and spreading are Rac to control lamellipodia and cdc42 to control filopodia (Burridge and Chrzanowska-Wodnicka, 1996). Indirect mechanotransduction is also able to alter cell fate. Extracellular signal-regulated kinase (ERK)/MAPK signaling can be a key modulator for both osteogenic and adipogenic phenotypes. Osteogenic topographies alter expression of ERK at both the genomic (Dalby et al., 2008) and proteomic (Xiao et al., 2002) levels in MSCs. ERK signaling controls nuclear transcription factors. One such transcription factor that has been linked to ERK is RUNX2 (Prusty et al., 2002), the osteogenic master gene that is essential for osteoblastic differentiation (Figure 2). ERK signaling also links into PPARγ, important for adipogenesis (Yang et al., 2008), stat1 and 3 implicated in induction/reduction of osteogenesis (Petersen et al., 2008).

For cells to adhere to a synthetic surface, the material has to replicate an ECM motif or absorb ECM proteins to promote cell attachment, therefore, cell survival, and subsequent function.

## Chemically Controlling Stem Cell Fate

Cells respond to chemical information on a surface in various ways. Most generally, cell adhesion depends on the hydrophobicity of the chemical structures on the surface. Surface hydrophobicity controls and directs the adhesion of serum proteins on the surface and, hence, the presentation of these proteins chemical groups can influence cell behavior. Simple surface functionality (inclusion of alcohols, amines, acids, for example) has been shown to influence stem cell fate (Curran et al., 2005, 2006). Remarkably simple chemical groups incorporated into a polymer hydrogel have been demonstrated to influence stem cell differentiation (Benoit et al., 2008) as discussed in more detail below. Surface functionalization with single amino acids (Rawsterne et al., 2007) showed systematic control of adhesion and spreading of fibroblasts that correlated directly with the logP of the surface bond amino acids. Since it is still not possible to rationally design polymers with chemical functionalities that control stem cell behavior, large arrays of polymers onto glass slides have been used to identify polymers with differentiation inducing potential (Tourniaire et al., 2006; Mei et al., 2010). The most effective ways to control cell–surface interactions involves bioconjugation with bioactive elements, such as short peptides or cell adhesive proteins (typically fibronectin) through techniques, such as soft lithography.

Soft lithography includes printing, molding, and embossing. It is advantageous as it results in defined and controllable surface chemistries (Qin et al., 2010), over a range of substrates, in an inexpensive manner (Kane et al., 1999). It has been an important step in the miniaturization process to create novel technology for both health care and biotechnology, e.g., lab on a chip and microfluidics. Microcontact printing is particularly relevant to biological systems (Gates et al., 2005), where it is possible to create adhesive and non-adhesive areas within a substrate to study cell–surface interaction (Kane et al., 1999). This technique achieves spatiotemporal control that allows creation of defined patterns of polymers, for example, synthetic polymers or natural proteins, such as fibronectin, and has not only been employed to study cell survival (Chen et al., 1997) but also cell differentiation. McBeath et al. employed this method to pattern fibronectin of differing areas (1024 and 10,000 μm^2^) stamped onto non-adhesive background. Confining cells to these adhesive areas showed that morphology and cell spreading was instrumental to differentiation. Specifically, MSC spreading on large areas of fibronectin aided osteoblastic differentiation, whereas smaller stamps facilitated a rounded morphology, encouraging lipid storage, and adipogenic phenotype (McBeath et al., 2004). Using this system, they demonstrated that osteogenic phenotype is tension dependent and mediated by the RhoA downstream effecter ROCK (McBeath et al., 2004). An eloquent update of this study using similar sized microcontact printed fibronectin stars and flowers illustrated that geometrical features control cell ability to form adhesions and, hence, control tension. Specifically, they illustrated sharp points to be more osteogenic that rounded curves (Kilian et al., 2010).

Dip pen nanolithography (DPN) is a method by which surface chemistry can be applied to a substrate on the nanometer scale with precision. Essentially, it involves the use of (an array of) very fine atomic force microscopy (AFM) tips, which can be inked with a suitable biomolecule, and then brought into contact with a surface where the ink is transferred to a nanoscale feature on the surface (Ginger et al., 2004). Surface chemistry can be defined by the user and encompasses organic molecules (thiols, amines, peptides, and oligonucleotides), polymers, and metal ions. DPN can be used in biomaterials engineering whereby functional molecules are printed in such an arrangement that stem cells can react and respond to. It has been shown that certain functional molecules can illicit distinct responses in MSC behavior (Curran et al., 2005, 2006). Curran et al. set out to optimize the spacing and presentation of dots of “chemistry” to manipulate MSC behavior by creating patterns of –CH_3_, –NH_2_, –CO, and –CO_2_H of 70 nm width in square or hexagonal array with varying distance of pitch. They found that functionalized –CH_3_ surface maintained stem cell markers in comparison to tissue culture plastic and gold surfaces. They also showed that NH_2_ dots can increase adhesion and osteogenesis (Curran et al., 2010).

Polymer pen nanolithography (PPL) is a “direct write” technique that uses soft elastomeric tip arrays to deliver inks/materials to the surface. PPL effectively combines the feature size control of DPN with large area capability of contact printing. The feature size also can be regulated by the amount of force applied to the elastomeric pen arrays and tip-substrate contact time. Mirkin et al. (Giam et al., 2012) aimed to define the relationship between feature size of a fibronectin coated area and stem cell fate. Fibronectin, patterned onto the substrate via PPL, direct the MSC differentiation toward an osteogenic pathway. In addition, Fibronectin substrate with nanoscale features (300 nm) are more effective in inducing osteogenic behavior than microscale feature size (1 μm).

An alternative method to incorporate a chemical component to a material is through the use of nanofibers. Nanofibers can be created in a variety of methods; phase separation, electrospinning, and self assembly, each with their own advantages and disadvantages depending on the application (Rim et al., 2013). The fibers can be made from a myriad of polymers or natural proteins and can be further modified by the addition of bioactive molecules. For example, Frith et al. conjugated RGD peptides to self-assembled poly(ethylene oxide) copolymers (PS-PEO) (Frith et al., 2012). Changing the ratio of the copolymer and polystyrene homopolymer creates defined spacing between PEO domains (34, 44, 50, and 62 nm) to which the functional group are tethered to, then seeded with MSCs. They found that spacing of 34 and 44 nm encouraged cell spreading, the cells formed larger (super mature) adhesions and when cultured in osteogenic media, promoted increase in alkaline phosphatase (ALP) expression. In comparison at 50 and 62 nm, cells remained rounded and under adipogenic conditions, oil red O staining was observed (Frith et al., 2012). That cells remained rounded until spacing was reduced to 44 nm might be that a critical size was needed to switch from adipogenic to osteogenic differentiation (i.e., a certain number of integrin need to gather) or from differences in affinity of RGD group used (e.g., low affinity linear RGD compared to high affinity cyclic RGD) (Kilian and Mrksich, 2012).

Using the electrospinning technique, it is possible to create composite fibers, i.e., polymer and bioactive compounds, such as gelatin (Zhang et al., 2005), hydroxyapatite (Lee et al., 2010), and demineralized bone powder (Ko et al., 2008). Ko et al. utilized the material not only as an *in vitro* culture method but also an *in vivo* scaffold that remained in a mouse model for 12 weeks (Ko et al., 2008). While capable of acting as a scaffold, cell infiltration is a concern. Bone formation was limited to the periphery of the construct, while the center was subject to hypoxic conditions. Porosity, therefore, remains an issue which, for future applications, must be balanced with the load-bearing properties.

The discovery of using simple chemical functional groups that direct MSC behavior by effectively controlling their differentiation potentials could lead to production of simple, cheap biomaterials for applications in regenerative medicine. Anseth et al., reported in 2008, the introduction of a small set of functional groups (with different charges, hydrophobicity) into PEG hydrogels and showed that they could induce MSC differentiation to different lineages (Benoit et al., 2008). These functional groups included –NH_2_, t-butyl, phosphate, –F, and –COOH. Depending on their charge and hydrophobicity, different MSC differentiation potential was observed, i.e., hydrophobic functional group like t-butyl induced adipogenic phenotype, while charged functional group (phosphate) promote osteogenic lineage and acid functionalized hydrogels demonstrated chondrogenesis.

Simple chemical groups are sufficient to influence MSC differentiation. Most likely these groups recruit and bind serum proteins in different ways thereby controlling presentation of adhesive groups. It will be of tremendous use if chemical functionality could be introduced in 3D scaffolds with precise spatial control. Ongoing developments in nanofabrication (both top down and bottom up) are likely to contribute significantly in the next decades.

## Cell Response to Topography

Cell interactions with topography were first noted by Harrison in 1911. The term contact guidance was later coined in the 1950s when it was reported that altering the appearance of a cell’s surroundings, in this case, density of fibrin networks resulted in changes in morphology of heart fibroblasts (Harrison, 1911; Weiss and Garber, 1952). Research in the area was popularized by Curtis and Wilkinson who applied development in microelectronics miniaturization to cell cultures through the 1980s and onwards (Wilkinson, 2004; Anderson, 2015). Thus, the term has long been employed to describe conformation to topography (Dalby, 2007). *In vivo*, the ECM topographical features are native to matrix infrastructure, their conformation provides the cells with behavioral cues. *In vitro*, it has been proven that the topographical cues influence stem cell behavior [altering gene expression that results in changes to adhesion, proliferation, and cytoskeletal conformation (Putnam et al., 2001; Dalby, 2007)], and it has been the work of many scientists to manipulate this interaction.

It has only recently been established that cells interact with their nanoscale environment, i.e., features much smaller than the cells themselves (Curtis et al., 2001; Dalby et al., 2002a; Gallagher et al., 2002). With the evolution of top-down lithography techniques, such as electron beam lithography (EBL) and aforementioned DPN (Curran et al., 2010), it is now possible to pattern areas large enough for cell experimentation (mm^2^–cm^2^) with features down to 10 nm in size (Gadegaard et al., 2003a). For a number of years, two separate approaches have dominated in topographical surface patterning: highly ordered patterns with sub-nm positioning error and random sub-μm roughened substrates. Ordered materials, typically generated by EBL, produce surfaces with low cell adhesion. By contrast, random sub-μm roughening can modify MSC differentiation relative to planar controls (Leven et al., 2004). Other topographical fabrication techniques include photolithography (Clark et al., 1987, 1990) and polymer demixing (Dalby et al., 2002b).

**Figure 3:**
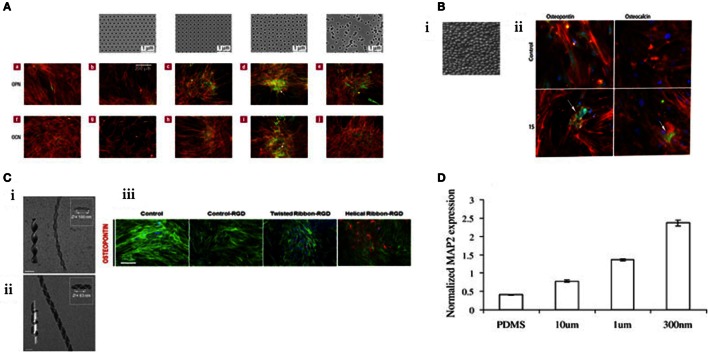
**MSC response to topographical features**. **(A)** EBL nanopatterned structures result in changes in gene expression. Those that exhibited “disorder” were found to stimulate osteogenesis. Reprinted (adapted) from Nature, copyright (2007) (Dalby et al., 2007). **(B)** i. Titanium was anodized to create a topography of 15 nm pillars. ii. These pillars were found to be osteoinductive by immunostaining for osteopontin and osteocalcin (green). Reprinted (adapted) from *Acta Biomaterilia*, copyright (2009) (Sjöström et al., 2009). **(C)** Creation of twisted nanoribbons at a periodicity similar to that of collagen (ii) resulted in osteogenic phenotype. (iii) Reprinted (adapted) from *ACS Nano*, copyright (2013) (Das et al., 2013). **(D)** MSCs can be differentiated toward a neural lineage using nanoscale channels, characterized by increasing MAP2 expression. Reprinted (adapted) from *Experimental Cell Research*, copyright (2007) (Yim et al., 2007).

Highly controlled-disorder patterns have been generated with EBL. In these systems, random and highly ordered cell environments were mimicked using 120 nm diameter (100 nm deep) pits with random placement of the features or fixed 300 nm centre–centre spacing in a square pattern. MSC growth substrates were also fabricated with deliberately disordered pits in a square arrangement (±20 and ±50 nm offset). While planar control, true square, and random substrates produced only negligible differentiation, bone differentiation was observed on the disordered patterns (Figure 3A) (Dalby et al., 2007) with similar efficiency to that obtained following soluble chemical (dexamethasone and ascorbate) treatment. Disordered systems can also be applied to other materials, such as titanium substrates that are of value clinically due to load-bearing properties (Sjöström et al., 2009, 2013). Anodising titanium using through mask templating with Ps-b-P4VP allows precision patterning in bulk for both 2D and 3D designs. Patterning pillars to a height of 15 nm is one such design that was found to be osteoinductive (Figure 3B) (McNamara et al., 2011).

Until recently, it has been considered that high precision manufacture (the top-down approach) is a requirement to gain precise control of the MSCs at the nanolevel. Nanofabrication engineers constantly strive to increase the complexity of designs that will be important for enhanced understanding of cell behavior at the nanoscale. Such criteria are readily met by EBL although the demand for scalability from current research level to that necessary in a clinical device (tens of square centimenter) may be a limitation of EBL due to the serial manner in which the patterns are produced.

Improving levels of order and disorder are becoming achievable with bottom-up methods, such as polymer phase separation (Affrossman et al., 2000), colloidal lithography (Denis et al., 2002), block copolymer lithography (Hur and Kim, 2002), and micelle lithography (Huang et al., 2009), where larger areas can be fabricated more simply. Block co-polymer micelles can, in fact, be generated with similar scale and level of order to the ±50 nm error EBL-fabricated pits (Krishnamoorthy et al., 2006) and have been shown to have osteogenic effects (Sjöström et al., 2009, 2012, 2013; McNamara et al., 2011; Maclaine et al., 2012). Furthermore, control of order to reduce any offset (rigid order of pitted features) has been shown to provide enhanced MSC growth that could be important, for example, provision of large numbers of high-quality stem cells (McMurray et al., 2011; Tsimbouri et al., 2012). The technology also appears to apply to other stem cell types, notably with ESCs where control of differentiation and growth have also been noted (Chen et al., 2012; Ji et al., 2012; Kingham et al., 2013; Kong et al., 2013).

One of the most attractive features of nanostructured surfaces as a tool for cell engineering is simplicity of mass production. Use of nickel shims (negative copies of the master structures made via electroplating) for embossing and injection molding allows high-throughput (incorporated within existing production lines) manufacture, with down to 5 nm fidelity, in a wide range of thermoplastics that could be used directly for cell culture (Gadegaard et al., 2003b).

Fabrication methods are ever evolving and engineering with complexity, creativity and replicating an aspect of nature. Das et al., inspired by the natural conformation of collagen, synthetically created Silica nanoribbons to mimic the *in vivo* cell–surface interaction to differentiate MSCs. The nanoribbons were synthesized using Gemini type amphiphiles to create two chiral nanoribbons with differing periodicities (measured as *D*) with either helical *D* = 100 nm (Figure 3C i) or twisted *D* = 63 nm confirmation (Figure 3C ii). MSC osteoblastic differentiation was upregulated when seeded on the twisted fibrils (Figure 3C iii) (Das et al., 2013). Notably, 63 nm periodicity is close to the 67 nm repeat pattern of collagen and, thus, is more representative of the natural bone environment noting that bone is >90% collagen (Dalby et al., 2014).

There are further illustrations that topography has been utilized to tune MSC differentiation beyond the conventional lineage repertoire – toward a neural lineage. The creation of channels prevents cell spreading and provides directionality. MSC expression of neuronal marker microtubule-associated protein 2 (MAP2) increased in response to nanoscale channels in comparison to microscale (Figure 3D) (Yim et al., 2007). Exploring the transition from microscale to nanoscale reveals the differential behavior of cells in response to scale of their environment.

## Alterations in Matrix Stiffness

Stiffness of the cell’s environment is relevant to all stages of development, from embryogenesis (Pouille et al., 2009) to terminal cell differentiation (DuFort et al., 2011). Changes in tissue stiffness can be indicative in certain disease states. For example, breast cancer tumors are more rigid than the surrounding tissue due to clusters of collagen fibrils, which increases matrix stiffness. As shown experimentally, mammary epithelial cells that have been cultured on compliant matrices behave normally whereas those cultured on stiffer materials invade the basement membrane disrupting tissue formation and promoting malignancy (Paszek et al., 2005; Wei et al., 2015). Therefore, alterations in stiffness have a direct result on phenotype (as discussed in *Cell–Surface Interaction*).

Hydrogels are the principal tool for investigating cell response to stiffness *in vitro*. They can be synthesized from an array of polymers (including biological polymers and peptides) where the degree of crosslinking can be tailored to alter stiffness properties as desired by the user. Due to their properties of compliance and high hydration, they can be utilized to mimic natural tissues. Mimicking the stiffness of a particular tissue type can guide cellular behavior toward a particular phenotype (Discher et al., 2005, 2009). It is further advantageous as hydrogels can be utilized as a delivery system for functional molecules. For example, using hydrogels for delivery of dexamethasone (Nuttelman et al., 2006), a synthetic corticosteroid that increases ALP secretion and bone morphogenetic protein (BMP), a signaling cytokine has been shown to drive osteoblast differentiation (Kim et al., 2007; Rahman et al., 2015). Furthermore, it is now possible to create hydrogel arrays that can test cell response to mechanical changes in a high-throughput manner (Gobaa et al., 2011).

**Figure 4:**
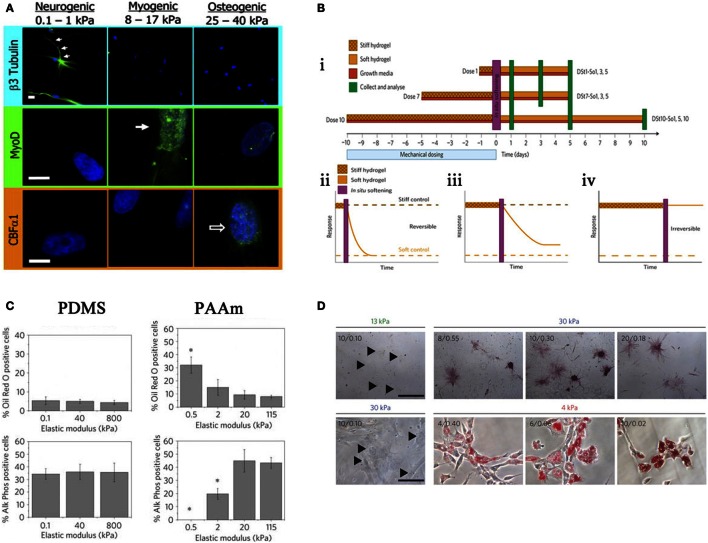
**Effects of stiffness**. **(A)** Altering stiffness results in differentiation into specified lineages, soft, intermediate, and hard matrices induce the differentiation of neuronal, myogenic, and osteogenic phenotypes, respectively. Reprinted (adapted) from *Cell*, copyright (2006) (Engler et al., 2006). **(B)** The effects of preculturing hMSCs on stiff substrates prior to seeding. ii. 1 day, iii. 7 days, iv. 10 days, where long-term pre-treatment resulted in maintenance of phenotype. Reprinted (adapted) from *Nature*, copyright (2013) (Gilbert et al., 2010). **(C)** Altering the elastic modulus resulted in same trend as **(A)** for PAAm substrates but not PDMS. Reprinted (adapted) from *Nature* ref copyright (2012) (Trappmann et al., 2012). **(D)** Altering the pore size of substrates does not result in changes in differentiation for that stiffness. Reprinted (adapted) from *Nature*, copyright (2014) (Wen et al., 2014).

In 2006, Engler and Discher produced a seminal study utilizing hydrogels to demonstrate that MSCs were responsive to a range of substrate stiffness, which in turn influenced differentiation. Three different substrate stiffnesses measuring 0.1–1, 8–17, and 25–40 kPa that represent E_brain_, E_muscle_, and E_bone_, respectively (where E is the elastic modulus), were compared. It was noted that cell morphology was altered in response to these different moduli and cells began to take the phenotype of the native cells of those tissues (Figure 4A) (Engler et al., 2006).

In 2010, Gilbert showed that a pre-culture of muscle stem cells (MuSC) on pliant materials aided self-renewal of MuSC *in vivo* (Gilbert et al., 2010); therefore, *in vitro* culture conditions are central to cell behavior *in vivo*. This has been described as “mechanical memory” and has recently been tested by Yang et al. MSCs were cultured on stiff substrates for differing times prior to seeding on a soft substrate (Figure 4B i), it was shown that there is a correlation between duration of pre-treatment and osteogenic phenotype. Those cells cultured the longest on stiff surface prior to transfer to a soft surface had a larger proportion of ALP positive cells and increase in Runx2 expression. With 10 days pre-treatment, osteogenic phenotype was maintained without the need for constant mechanical stimulation (Figure 4B ii–iv). Furthermore, this can be done *in situ* by culturing on light responsive hydrogels, after irradiation at 365 nm for 360 s the hydrogel changes from stiff (~10 kPa) to soft (~2 kPa) (Yang et al., 2014).

It is known that cell spreading and morphology is important for differentiation (Matsuoka et al., 2013). Khetan et al. investigated this using either permissive (degradable) or inhibitory (undegradable) hydrogels. These gels are phototunable, when exposed to light the gels are degraded allowing cells to remodel and deform the matrix. Degradable hydrogels encouraged spreading of MSCs and, therefore, osteogenesis, whereas restriction of spreading by crosslinked hydrogels maintained cellular circularity and, therefore, stimulated adipogenesis (Khetan et al., 2013). But is morphology the defining factor? Huebsch et al. confined murine MSC to pores in RGD modified hydrogels and, therefore, morphology was maintained rounded, traditionally a prerequisite for adipogenesis. Encapsulated cells still responded to matrix elasticity as 22 kPa stimulated osteogenesis even with rounded morphology. This was related to traction (the force generated by cytoskeletal pulling on the substrate) the cells were able to deform the substrate to gather RGD ligands, creating traction or high intracellular adhesion. Taken together, the results show that the presentation of ligands to the cell is crucial for creation of adhesion and subsequent traction generated by matrix reorganization is central to driving the osteogenic phenotype (Huebsch et al., 2010).

Stiffness is assumed to define the bulk properties of a material. However, different materials of comparable Young’s Moduli can illicit differing responses in MSCs, for example, PDMS and PAAm gels as depicted in a study by Trappmann et al. (2012). Here, MSCs were seeded on PAAm at a range of elasticities similar to that Engler et al. demonstrated in 2006 (Engler et al., 2006). PAAm also followed the same trend, i.e., osteogenic phenotype at a high elastic moduli (stiff), adipogenic phenotype at low elastic moduli (soft). Conversely, PDMS at the same range of moduli showed no trend (Figure 4C). Further experimentation revealed that both materials had a comparable bulk stiffness, however, topography (pore size) was altered. They argue that ECM adsorption differed with topography as the ECM dictated the number of anchoring points available to the ECM. Cells, therefore, respond to the mechanical properties of the ECM rather than the bulk stiffness of the substrate. However, in a more recent study, Wen et al. argue that differentiation occurs regardless of protein tethering and that stiffness is the deciding factor. Adjusting the crosslinking density of their hydrogels to alter pore size for each stiffness, suggested that pore size had no effect on the differentiation of cells; as pore size varies, phenotype is maintained (Figure 4D). In short, it is cell deformation of the material that is driving the process (Wen et al., 2014).

## How Do Cells Process This Materials Information? The Central Role of Adhesion

In his 2005 commentary, Ingber discusses that tissue organization must be controlled by other factors in addition to soluble morphogens and local tissue factors. He explains how biochemical reductionism tends to overlook factors, such as tension, and instead focuses on genes and gene products (Ingber, 2005). While we consider his ideas of tensegrity (Ingber, 1993) to be beyond the scope of this article, Ingber’s tensegrity model shows that the adhesions are the “tent-pegs” to which the cellular guy ropes (the cytoskeleton) are attached to give the cell the pre-stress required for stability, development of tension and possibly tensegrity. Through this mechanism, tension directly links to cell proliferation, functionalities, and differentiation.

Changing environmental factors culminate in relatively similar cellular detection, integrin binding, and response. However, there is differing cellular response to the microscale in comparison to the nanoscale. At the microscale, it is easy to envisage how cells are forced to contact guide (align) by features of a similar height to themselves. At the nanoscale, the cell will be guided one adhesion at a time. As adhesive proteins encounter a nanoscale cue (e.g., a nanogroove), the cells adhesions will remodel along the cue, reorganizing the cytoskeleton and direction of tension applied to the adhesion (Teixeira et al., 2006). Adhesion to a material begins with the rearrangement of actin filament to form microspikes, or filopodia, that have been shown to interact with features as small as 10 nm (Dalby et al., 2004). This contracture will gather integrins forming a large adhesion and will group actin filaments into stress fibers, a cell activity important for cell survival and exquisitely modulated by force (Riveline et al., 2001; Jiang et al., 2003). Changes in chemistry, stiffness, and topography influence the size and number of cell adhesions. Furthermore, it seems likely that there are critical adhesion sizes for cells to be able to gather spatial information through filopodial extension (Arnold et al., 2008). Adhesions can be classified by size and include focal complexes (<2 µm long, transient, involved in motility) FAs (>2 µm long, stable, formed during cell maturation and ECM production) and super mature adhesions (SMAs that are very large >5 µm long). The currently used classification of SMAs is really an evolution of the classical “dot” and “dash” adhesions described by Bershadsky et al. (1985).

The study of FAs appears to demonstrate that alterations in the size and number of adhesions are important for MSC differentiation. As has been discussed, MSCs differentiate to bone efficiently on a disordered nanoscale pattern (Dalby et al., 2007). Investigation of adhesion size has demonstrated that on the osteogenic pattern much larger adhesions were generated by the MSCs (Dalby et al., 2007). It was postulated by the Spatz group that the disordered nature allowed adhesive points to group closer together [within the critical 70 nm range described by Cavalcanti-Adam (Albelda and Buck, 1990; Cavalcanti-Adam et al., 2008)] and, thus, facilitate integrin gathering (Kingham et al., 2013) and the formation of SMAs (Biggs et al., 2008, 2009) (> 5 µm long). It is likely that these larger adhesions are stabilised by scaffolding proteins such as RACK1 (Buensuceso et al., 2001; Dalby et al., 2008), decreasing cell motility, but allowing formation of cytoskeletal tension (Balaban et al., 2001; Shemesh et al., 2005) important to MSC fate culminating in large morphology and osteogenic phenotype (Curtis et al., 2001; Putnam et al., 2001; Wen et al., 2014).

There is a variation in size of mature cell types generated from MSCs (small, round adipocyte to large orthogonal appearance of the osteoblast). We can postulate a role for the natural environment of the stem cell in defining cell morphology. This environmental regulation of adhesion size, cytoskeletal tension, and overall cell morphology will have important roles in the induction of cell differentiation, which importantly can be dictated by designing the material interface. The ability to control cell fate through presentation of chemical functionality (i.e., promoting the binding of transmembrane integrins to ECM proteins or peptide ligands) is well understood. The roles of stiffness and topographical interventions are less intuitive. With regard to material stiffness, it has been observed that FA size is increased on materials of higher stiffness, which enhances the ability of a cell to form a contractile cytoskeleton (Wen et al., 2014; Yang et al., 2014). These cytoskeletal changes determine cell tension, morphology, and fate.

## A Dynamic Future?

All the examples that have been discussed thus far have been “static” in nature, i.e., a single topography or chemical functionality is used to perform a specific role (self-renewal or differentiation). However, the stem cell niche is dynamic, regulating growth and differentiation on demand. Thus, it makes sense that next-generation materials should also have dynamic aspects, in particular to support self-renewal *and* differentiation with spatiotemporal control. Indeed, stimuli-responsive surfaces have attracted significant scientific interest in recent years in this context. Stimuli, such as light (Ohmuro-Matsuyama and Tatsu, 2008; Petersen et al., 2008; Liu et al., 2009; Wirkner et al., 2011a), enzymes (Todd et al., 2007; Zelzer et al., 2012), temperature (Yamato et al., 2002), and electric fields (Yeo et al., 2003), have been investigated. These external stimuli should ideally be cytocompatible and bioorthogonal in order for them to be utilized in a cellular context.

Light has been applied to control cell adhesion, typically by changing the chemical functionality or presentation of RGD molecules. In 2008, Del Campo et al. demonstrated the modification of RGD with a photoresponsive caging group on the carboxylic acid side chain of aspartic acid. Prior to irradiation, the photocaging group prevents integrin recognition and consequent adhesion. In response to light, the caging group is released allowing for on-demand adhesion. They concluded that this system had many applications and suggested developing patterned areas of photoactivity (Petersen et al., 2008). In a follow-up study, they modified the photoliable element to include a 4,5-dialkoxy 1-(2-nitrophenyl) ethyl that was incorporated in between the amine terminated surfaces and RGD peptide. The photoliable element could be irradiated to allow adhesion and in addition they also patterned the substrate demonstrating specific area of HUVEC (human umbilical vein endothelial cell) attachment (Wirkner et al., 2011b). One of the main advantages of using light as a method of controlling adhesion is that it can be applied locally, e.g., using photomasks. This has enabled patterning of a cell culture dish with spatiotemporal control permitting adhesion in defined areas on demand. The examples that have been discussed so far relate to activation/deactivation by making use of one-off breaking of chemical bonds. In order to produce a reversible system, Jiang et al. used azobenzene as a conformational switch to alter the presentation of RGD ligands. Irradiation at 340–380 or 450–490 nm resulted in trans-cis or cis-trans isomerization, respectively, either promoting adhesion or preventing cell adhesion to substrate (Liu et al., 2009).

Photochemical control can also be applied in three-dimensional systems. In 2009, Anseth et al. utilized copper free click chemistry to synthesize hydrogels with thiol-ene groups that could be photocoupled in order to pattern biochemical functionalities at user-defined locations. They showed the surface to maintain a population of 3T3 fibroblast cells (DeForest et al., 2009). Later, DeForest and Tirrell improved such a system creating reversible patterning of bioactive ECM protein (i.e., vitronectin) inside a three-dimensional polymeric hydrogel scaffold. In doing so, they succeeded in differentiating hMSCs to osteoblasts.

Mosiewicz et al. employed both light and enzymatic control of substrate. Caged FXIIIa (transglutaminase factor XIII) was covalently incorporated into a PEG hydrogel. FXIIIa enzyme catalyzes reactions between ε-amine of lysine with γ-carboxamide residue of glutamine. Upon exposure to light, the caged substrate in the hydrogel activated and FXIIIa catalyzed reaction of the substrate with counter-reactive substrate of biomolecule in a covalent fashion within the hydrogel matrix. Through this photopatterning of hydrogel with desired biomolecules 3D manipulation of MSCs within hydrogel matrix can be achieved spatiotemporarily (Mosiewicz et al., 2013).

Electric field has been used as a stimulus by Mrksich et al. Incorporating an electroactive moiety, *O*-silyl hydroquinone on the surface with RGD at a defined electric field (550 mV), *O*-silyl hydroquinone undergoes electrochemical oxidation to form benzoquinone, thereby hydrolyzing the silyl ether that causes the selective release of RGD ligand from the surface (Yeo et al., 2003).

Stimuli such as light and electric field are unsuitable for some biological applications. Enzymes, however, provide an alternative. Enzymes act as a benign physiological trigger with the potential advantage of selectivity, specificity, biocompatibility, and dynamicity, and perform under physiological environment (Hedstrom, 2010). Enzymes are potentially an effective alternative to trigger a chemical change in the surface that can affect MSC behavior. Until now, there are very few examples of enzyme responsive surfaces in the literature. To create such a platform, Todd et al. utilized solid phase peptide synthesis (SPPS) to tether amino acids to a glass coverslip. The advantage of SPPS is that any sequence can be synthesized depending on the application. Todd et al. developed a sequence Fmoc-A↓ARGD-Glass that is cleavable by the enzyme elastase. The full sequence, Fmoc capped, prevents cell adhesion to RGD. Application of the enzyme cleaves the sequence at the dialanine linker allowing attachment to RGD. This system is biocompatible and easily controlled (Todd et al., 2007; Zelzer et al., 2012).

## Summary

At present, these materials approaches have characterized the nature of MSC adhesion and subsequent behavior. What is lacking is an optimal system that provides the quantity of stem cells required for a TE construct. Furthermore, material use as *in vivo* scaffolds is still not fully exploitable due to limited invasion, porosity, vascularization, and load-bearing properties that are all challenges that have still to be optimally addressed. The key to delivery of regenerative therapies lies in the development of stem cell culture platforms where stem cells can grow and differentiate into different phenotypes for incorporation into TE scaffolds supported by biomaterials. Materials have been used to demonstrate basic niche functions. However, the current materials strategies, although providing new insights into stem cell biology, especially, MSC behavior, are static technologies and have certain limitations. The materials available to date that target continued self-renewal are useful for promoting growth but they are poor in differentiation and *vice versa*. The immediate challenge is to fabricate niche-mimicking biomaterials, i.e., a material system where MSCs will be cultured as a growing stem cell population and when triggered (either user induced or autonomously, by cell secreted factors), will switch to a phenotype of choice, on demand.

## Author Contributions

All authors were involved in writing the review: HA, Univeristy of Glasgow. MD, University of Glasgow. JS, University of Strathclyde. RU, CUNY.

## Conflict of Interest Statement

The authors declare that the research was conducted in the absence of any commercial or financial relationships that could be construed as a potential conflict of interest.
